# In-depth sequencing of the siRNAs associated with peach latent mosaic viroid infection

**DOI:** 10.1186/1471-2199-11-16

**Published:** 2010-02-16

**Authors:** François Bolduc, Christopher Hoareau, Patrick St-Pierre, Jean-Pierre Perreault

**Affiliations:** 1 RNA group/Groupe ARN, Département de biochimie, Faculté de médecine et des sciences de la santé, Université de Sherbrooke, Sherbrooke, QC, J1H 5N4, Canada

## Abstract

**Background:**

It has been observed that following viroid infection, there is an accumulation of viroid-derived siRNAs in infected plants. Some experimental results suggest that these small RNAs may be produced by the plant defense system to protect it from infection, indicating that viroids can elicit the RNA-silencing pathways. The objective of this study is to identify in the peach latent mosaic viroid (PLMVd), a model RNA genome, the regions that are most susceptible to RNA interference machinery.

**Results:**

The RNA isolated from an infected tree have been used to sequence in parallel viroid species and small non-coding RNA species. Specifically, PLMVd RNAs were amplified, cloned and sequenced according to a conventional approach, while small non-coding RNAs were determined by high-throughput sequencing. The first led to the typing of 18 novel PLMVd variants. The second provided a library of small RNAs including 880 000 sequences corresponding to PLMVd-derived siRNAs, which makes up 11.2% of the sequences of the infected library. These siRNAs contain mainly 21-22 nucleotide RNAs and are equivalently distributed between the plus and the minus polarities of the viroid. They cover the complete viroid genome, although the amount varies depending on the regions. These regions do not necessarily correlate with the double-stranded requirement to be a substrate for Dicer-like enzymes. We noted that some sequences encompass the hammerhead self-cleavage site, indicating that the circular conformers could be processed by the RNA-silencing machinery. Finally, a bias in the relative abundance of the nature of the 5' nucleotides was observed (A, U >> G, C).

**Conclusions:**

The approach used provided us a quantitative representation of the PLMVd-derived siRNAs retrieved from infected peach trees. These siRNAs account for a relatively large proportion of the small non-coding RNAs. Surprisingly, the siRNAs from some regions of the PLMVd genome appear over-represented, although these regions are not necessarily forming sufficiently long double-stranded structures to satisfy Dicer-like criteria for substrate specificity. Importantly, this large library of siRNAs gave several hints as to the components of the involved silencing machinery.

## Background

Viroids are small (240-460 nucleotides, nt), single-stranded circular RNA pathogens that self-replicate in the plants causing important economical losses in agricultural industries [1]. Their genomes do not encode any protein, so they rely exclusively on host proteins. They are divided into two families: the *Pospiviroidae *and the *Avsunviroidae*. The members of the first family, for which the typical representative is the potato spindle tuber viroid (PSTVd), share a conserved central region, a nuclear localization and an asymmetric rolling circle replication that appears to involve host enzymes. Members of the *Avsunviroidae*, including the peach latent mosaic viroid (PLMVd), which is the viroid model in the present study, have been shown to accumulate in the chloroplasts [2]. The latter viroids possess hammerhead self-cleaving RNA motifs and replicate according to a symmetric rolling circle mechanism.

Plants accumulate a multitude of small non-coding RNAs (i.e. 18-25 nucleotides in length). The actions of most of these small RNAs result in gene repression through what is called post-transcriptional gene silencing (PTGS) to protect the plants from biotic stresses, like viroid or virus infection. Besides their role in response to pathogens, these small RNAs are also implicated in development and genome integrity [3]. Based on the detection of small RNAs, it has been established that viroids induce PTGS [4-8]. In addition, it has been demonstrated that a small hairpin derived from PSTVd was sufficient to cause symptoms in its natural host, providing a potential explanation for the pathogenicity mechanism [9]. Moreover the compact viroid structure makes them resistant to RISC degradation [10] at least in the case of PSTVd. However, contradictory reports have shown that, under some circumstances, viroid-derived small RNAs could protect the plants from viroid infection [11,12], making the association between RNA silencing and pathogenesis questionable.

In the case of PLMVd, some aspects of the RNA-silencing mechanism, including how it might trigger Dicer-like enzyme activity, were studied using a cell-free wheat germ assay [13]. In addition, we recently reported the results of a sequencing effort directed towards the siRNAs associated with PLMVd infection in peach leaves [14]. Specifically, a fraction of small, non-coding RNAs (ncRNAs) was isolated, cloned and sequenced according to the classical procedure for ncRNAs, and any PLMVd-derived siRNAs that were found were then analyzed. This work yielded several interesting observations, including the fact that PLMVd strands of both polarities may enter the peach RNA silencing pathways. These observations should be considered with caution as they are based on the analysis of only 60 PLMVd-derived siRNAs, which out of 284 ncRNAs detected in infected plant leaves, corresponds to only 3.9× coverages of the viroid genome. Moreover, since RNA silencing in plant includes a phenomenon of amplification by an RNA-dependent RNA polymerase [3], it was surprising to note that some regions of the genome were not covered by siRNAs.

High-throughput sequencing (HTS) is a method that provides a relatively unbiased and direct digital readout of cDNA sequences generated from an RNA sample [15,16]. The unparalleled ability of HTS to yield quantitative information on transcript abundance should help to give a more representative picture of the PLMVd-derived siRNA population than what is available right now. With the goal of obtaining a clearer view of the PLMVd-derived siRNAs, the sequencing of the small RNAs associated with this infection was repeated using HTS. Concurrently, the viroid species present in RNA samples used were also sequenced. The parallel sequencing of the viroid and derived siRNAs retrieved within a unique RNA sample should remove potential bias.

## Results and Discussion

**Figure 1:**
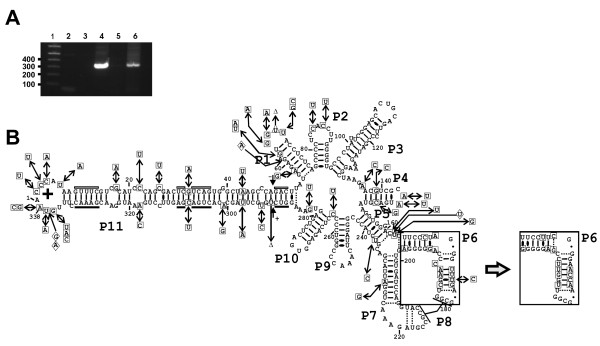
**Analysis of PLMVd replication and sequences**. (A) Agarose gel electrophoresis of RT-PCR amplifications performed in order to detect the presence of PLMVd in the RNA isolated from both the healthy and the infected leaf RNA samples. Lane 1 is a 1 kb-plus ladder that serves as a size standard. Lanes 2 and 3 are negative control reactions for the reverse transcription and PCR amplification, respectively performed in the presence of water alone. Lane 4 is a positive control PCR reaction performed using pPD1 plasmid that contains a dimer of PLMVd sequences as template. Finally, lanes 5 and 6 are the RT-PCR reactions performed in the presence of the RNA samples isolated from both the healthy and the PLMVd-infected samples, respectively. (B) Secondary structure according to previous studies [18] and nucleotide sequence of PLMVd novel variants. The (+) polarity sequence used to fold the above structure is the one that was detected twice following sequencing [NCBI accession number GQ499310]. All of the mutations found in the novel sequences are represented by the boxes around the structure. The mutations are identified by squares, the deletions by triangles and the insertions by diamonds. The alternative P6 stem found in one clone [NCBI accession number GQ499317] is illustrated in the inset. The arrows juxtaposed to the + and - signs identify the hammerhead self-cleavage sites of each polarity, while the boxes identify the conserved nucleotides that form the hammerhead motifs. The white objects indicate minus (-) polarity, while those in black denote the plus (+) polarity.

Total RNA was isolated from the leaves of one healthy and one PLMVd-infected tree. PLMVd infection was confirmed in the leaves of the infected tree by RT-PCR amplification, and could not be detected in the sample taken from the leaves of the healthy tree (Figure 1A). Subsequently, the DNA PCR products derived from the PLMVd amplification of the sample taken from the infected tree were isolated, cloned and sequenced. Eighteen different PLMVd sequences, varying from 337 to 339 nt in size [NCBI accession numbers: GQ499305 to GQ499322; also see Additional file 1], were obtained from the nineteen clones that were retrieved. These new sequences will be used in subsequent pairwise sequence comparison for the analysis of the PLMVd-derived siRNAs. All 18 sequences appeared to be new variants when they are compared to the 349 variants that have been reported to date in public libraries [17]. The sequence found twice was arbitrarily used for the representation depicted in figure 1B, and all mutations are reported in reference to this variant. This variant was folded according to the biochemically characterized secondary structure for another PLMVd variant [18]. With the exception of minor local rearrangements, it appears to be a good fit to the proposed secondary structure. Moreover, base pair covariations were observed at several positions, including several consecutive positions of the P6 stem in one variant (see Figure 1B, right inset). A total of 53 mutations, including substitutions of all types, deletions and insertions were detected throughout the PLMVd genome. Interestingly, in spite of the relatively important number of mutations observed in the P1 and P11 stems (26 out of the 53 mutations), all nucleotides critical for hammerhead self-cleavage activity were perfectly conserved. More importantly, all of the hammerhead sequences of both the plus (+) and the minus (-) polarities preserved the ability to fold into an active secondary structure due to the fact that base pair covariations occurred within the three critical stems that surround the catalytic core (data not shown). This observation provides a strong indication that the sequences obtained are derived from PLMVd species that possessed the ability to replicate, and not simply from dead-end products containing aberrant mutations.

Concurrently, the small RNAs (18-30 nt) present in the RNA sample above were isolated after fractionation on a 15% denaturing polyacrylamide gel. After the ligation of 5' and 3' adapters, these RNAs were amplified by RT-PCR. After purification on a 10% polyacrylamide gel, the resulting DNA samples were high-throughput sequenced using the Illumina Solexa technology. Reads that did not contain the 3' linker, those shorter than 16 nt, and those with ambiguous nucleotides were discarded so as to reduce, as much as possible, any further bias or wrong identification. This trimming steps yielded libraries of 12 476 493 and 7 862 905 total reads for the healthy and the PLMVd-infected samples, respectively (see Table 1). As a quality control, sequence homology searches were performed using the Exonerate software [19] allowing for no mismatches with *Arabidopsis thaliana *rRNA since the *Prunus persica *genome sequence remains incomplete (see Table 1). Nuclear, plastid and mitochondrial rRNA sequences were retrieved, indicating that RNA from the three compartments had been isolated. The rRNA fractions present in the healthy and the PLMVd-infected sequence collections were limited to only 1,2% and 4,5%, respectively (Table 1). These are relatively low levels of rRNA, and provide a good indication of the samples' quality [20].

**Table 1:** Analysis of the HTS data for the RNA obtained from both the healthy and the PLMVd-infected peach leaf RNA samples.

Description	HTS libraries	
	**Healthy**	**PLMVd-infected**
	
Unfiltered total reads	38 035 624	10 502 809
Total reads	12 476 493	7 862 905
Reads homologous to rRNAs	152 911	351 657
Mitochondrial	1 593	2 667
Nuclear	124 142	324 744
Plastid	27 176	24 246
Reads homologous to tRNAs	37 977	24 278
Reads homologous to PLMVd	2	884 234
PLMVd (+) polarity	1	423 951
PLMVd (-) polarity	1	460 283

**Figure 2:**
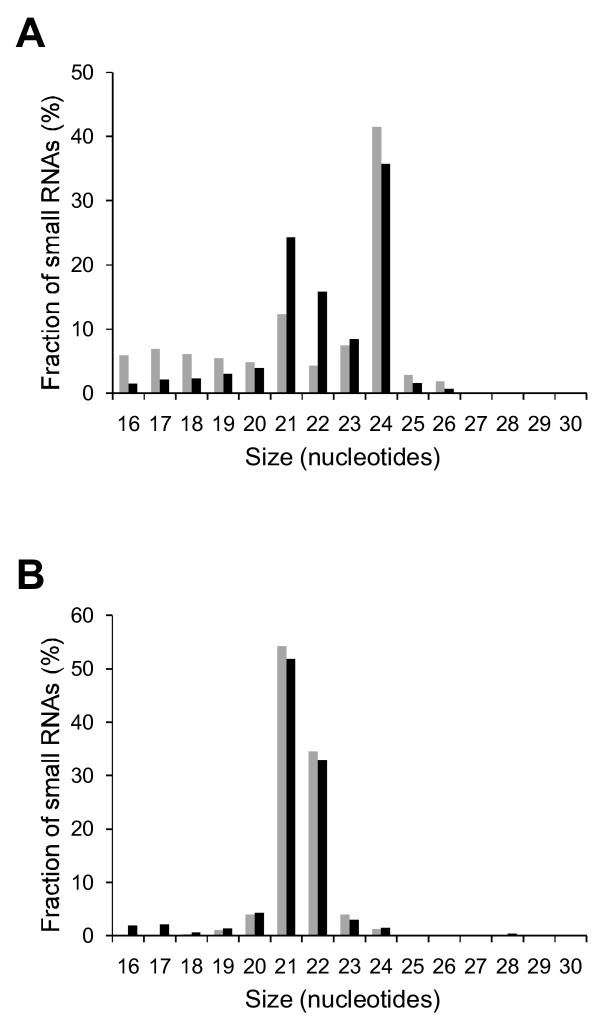
**Size fractionation of small RNA libraries**. (A) Total reads from both healthy (grey) and PLMVd-infected (black) peach leaf RNA librairies are classified according to their size in nucleotides. (B) PLMVd associated siRNAs from both the (+) (grey) and (-) (black) polarities are classified according to their sizes.

The RNA sequences were analyzed as a function of their size (Figure 2A). The most abundant small RNAs were those of 24 nt corresponding to DCL3 products [21] and described as heterochromatic siRNAs [22], followed by those of 21 and 22 nt, as has been previously observed in other plant species [23]. Importantly, a significantly higher amount of the 21 and 22 nt ncRNAs were observed when the PLMVd-infected sample was compared to that derived from the healthy leaves. Specifically, if we combine the 21-22 nt fraction into one, it increases from 16.6% in the healthy to 40.1% in the infected samples, representing a 2.4 fold increase (i.e. 141%), showing that the presence of PLMVd clearly increases these siRNAs populations.

Subsequently, both libraries were analyzed for the presence of PLMVd-derived sequences via a homology search using the novel variant viroid sequences. Only 2 reads, corresponding to only 16 × 10^-5 ^% of the total number (see Table 1), from the library derived from the healthy sample were found to be homologous with PLMVd sequences. This low number simply represents the background noise. Conversely, 884 234 sequences from the library derived from the infected sample were found to be homologous with the eighteen PLMVd sequences. This abundance of PLMVd-associated small RNAs corresponds to 11.2% of the reads of the infected library, and is significantly greater than what is observed for two *Pospiviroidae *family viroids (i.e. <5% for both the PSTVd [10] and the citrus exocortis viroid (CEVd) [24] that are known to accumulate in the nucleus). Among the sequences complementary to PLMVd, no apparent polarity bias was detected (i.e. (+) 423 951 and (-) 460 283). The relative abundances of the PLMVd replication intermediates of both polarities present in peach cells was previously estimated to be almost identical in a Siberian C cultivar (i.e. 2.2 ng and 2.0 ng for the (+) and (-) polarities respectively, per mg of tissues [2]. This is the same order of magnitude, allowing for experimental error, as was previously suggested for other PLMVd variants in a distinct peach cultivar [14]. This result confirms that if the PLMVd genome or its replicative intermediates, enter the plant siRNA pathways, both strands are susceptible to be implicated.

Relatively few species larger than 23 nt in size (Figure 2B) were found in the PLMVd-associated sequence subset. In fact, most of the reads were either 21 or 22 nt in size (765 766 sequences, representing 86.6% of all of the PLMVd derived sequences). Since Dicer-like enzymes cleave double-stranded RNA structures, it is tempting to assume that the viroid RNA genome (or replication intermediates) can be substrates of those enzymes *in vivo *as shown for PSTVd [10]. Moreover, *in vitro *studies have shown that incubation of PLMVd RNA generates siRNAs in a cell-free wheat-germ extract [13]. According to previous data using a genetic approach in *Arabidopsis thaliana *[21], more than one Dicer-like enzyme can produce siRNAs of 21-22 nt, rendering difficult and premature the identification of which one is involved with PLMVd silencing.

**Figure 3:**
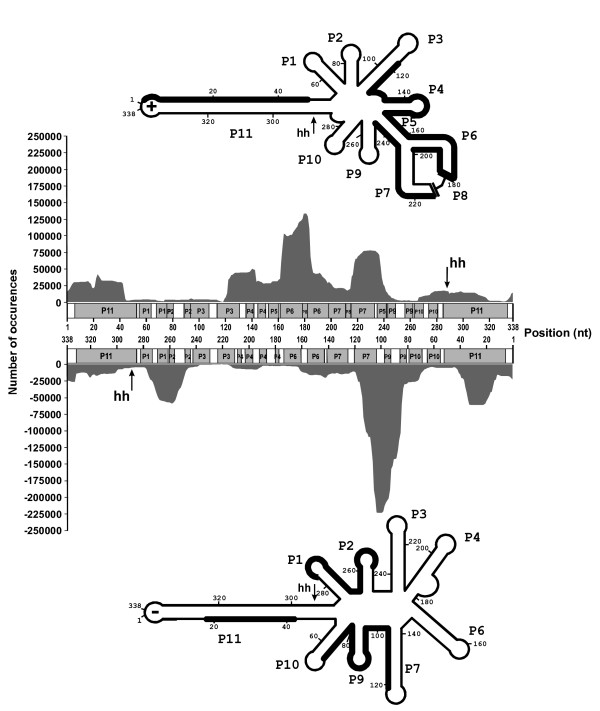
**Localization of PLMVd-associated siRNAs**. The number of occurrences of each nucleotide, when all of the aligned siRNAs are considered, is plotted over the nucleotide's position on the linear representation of the PLMVd genome. The upper graph corresponds to the PLMVd-associated siRNAs of (+) polarity, and the lower one to those of (-) polarity. Paired regions of the secondary structures (P) are shown in grey, while the single-stranded regions (bulges and loops) are shown in white. The letters "hh" indicate the position of the hammerhead self-cleavage sites. The regions of the secondary structures that have more than 25 000 occurrences are shown in bold on the proposed secondary structures for both the (+) and the (-) polarities. The secondary structures used are according to [18] and [25] respectively.

The alignment of these siRNAs along the PLMVd genome showed that it was covered in its entirely, but that strong concentrations of siRNAs were found in specific regions (Figure 3). For the (+) polarity, the siRNAs appeared to predominantly concentrate on the upper strand of the P11 long stem in addition to a region covering from the P3 to the P7 stems. These observations are partially in agreement with the previous analysis based on a small subset of siRNA sequences [14]. In this earlier case, the most abundant regions were found to be one strand of the P2 stem, the P3 stem and one strand of both the P7 and the P11 stems. With respect to the (-) polarity, the previous analysis did not permit the identification of the regions corresponding to the most abundant siRNAs [14]. The results presented here show that the siRNAs seem to be significantly more abundant in the P9 stem-loop region, and include fragments of both the P7 and P10 stems, in addition to part of one strand of the P1 stem, the P2 stem and one strand of the P11 stem (i.e. the lower strand). Together the differences observed here clearly justify this new HTS-based work.

Clearly, the accumulated siRNAs differ significantly for the two PLMVd polarities. The secondary structures adopted by the PLMVd strands of both polarities [18,25] differ, and may have an important impact on this phenomenon. It is likely that the accessibility of the double-stranded structure to the Dicer-like enzyme is an important criteria to consider. Surprisingly, both the P6 and P7 stems of the (+) polarity, which interact through the P8 pseudoknot that should reduce their accessibility to the enzyme, appear to constitute a preferred region as evidenced by the high abundance of siRNAs derived from this region. Also the P11 stem is a long double stranded RNA structure that could satisfy the Dicer-like enzymes for cleavage; yet, our results show that it is not necessarily the case. For example, only one strand of the P11 stem of both polarities was observed to preferentially accumulate siRNAs, while the other did not. One possible explanation might be that host proteins interact with the latter strand, thereby preventing Dicer-like binding. Interestingly, it is not the same strand of the P11 stem of both polarities that shows preferential accumulation. The reason for this phenomenon remains obscure. Moreover, it has been reported that disease symptoms induced by the hop stunt viroid (HSVd) in *Nicotina benthamiana *were independent of viroid accumulation levels, and instead relied on the involvement of the RNA-dependent RNA polymerase 6 (RDR6) [26]. Based on this, it is possible that Dicer plays a relatively important role, but not as important as RDR6 potentially could, and that the over-represented regions in the pool of PLMVd derived small RNAs may actually reflect the action of RDR6 and other as yet unknown factors more than the activity of Dicer-like proteins. If this is indeed the case, we can speculate that the viroids may potentially exert their pathogenicity through the tasiRNA pathway involving RDR6 and DCL-4, as it has been previously hypothesized [27]. Another aspect of the problem might be the preferential degradation of some siRNAs by the cellular machinery

Recent reports have shown that the sorting of small RNAs into *Arabidopsis *argonaute (AGO) complexes is influenced by the 5' terminal nucleotide [28-30]. In order to attribute the potential implication of specific AGO complexes, the nature of the 5' nucleotide of PLMVd-derived siRNAs of 21-22 nt was analyzed. PLMVd-derived RNA of 21 nt in size contain 48.7% of the time a U at the 5' end and 24.9% a C (i.e. C + U = 73.6%). Similarly, the PLMVd-derived siRNA of 22 nt harbor a U in the 5' end 39.4% of the time and a C in 29.2% (C + U = 68.6%). When the fraction of PLMVd-derived siRNA was removed from the library and the same analysis was repeated, the same bias for U and C was observed (C + U = 70.3% (19.0% + 51.3%) and 63.7% (23.1% + 40.6%) for the 21 and 22 nt ncRNA in size, respectively; data not shown). Conversely, the analysis of the library of small non-coding RNA obtained from the healthy leaves revealed a different bias for the nucleotide identity retrieved at the 5' end. The small RNAs of 21 as well as 22 nt in size showed a significant preference for a A and G (C + U = 43.0% and 36.8% for the 21 and 22 nt ncRNA in size, respectively, data not shown). In the latter case, the A was the most over-represented nucleotide in 5' (~ 40%). AGO1 and AGO5 were shown to preferentially recruit small RNAs beginning by a U and a C, respectively [29,30]. Taking into account this demonstration, the bias towards a U or a C in the 5' terminal nucleotide suggests that PLMVd-derived small RNAs, if they are implicated in the plant RNA silencing pathways, may be recruited by AGO1 and AGO5, if homologous protein complexes exist in the *Prunus *genus. More generally, it is tempting to suggest that the active AGO proteins in the infected plant cell differ from those retrieved in the healthy ones. However, this does not exclude that other AGO proteins may be involved in the binding of those small RNAs since plants may encode up to ten different AGO proteins with redundant functions [29].

Lastly, in the previous analysis, the use of a minimal set of siRNAs [14], led to the proposal that a potential bias exists for the transportation of the circular conformers into the cytoplasm where they trigger further RNA silencing. This hypothesis is based on the observation of a slightly larger proportion of siRNAs that overlapped the self-cleavage site of PLMVd, a species that is by definition derived only from circular conformers. According to the alignment illustrated in Figure 3, this is no longer the case. The regions that include the hammerhead self-cleavage sites are not part of the hotspots on the structure. However, PLMVd-derived siRNAs of both polarities are retrieved from the infected plant. Also, no RNA silencing components within the chloroplast have been described so far. These components are localized mainly in the cytoplasm. So the viroid needs to be transported into that compartment to be processed by the RNA silencing machinery. These results suggest that, at least, the circular PLMVd conformer of both polarities is transported into the cytoplasm from the chloroplast. This might also be the case for the linear monomers that are the most abundant replication intermediates [2]. Importantly, this study clearly shows that any conclusion based on a relatively small number of sequences should be taken with considerable caution.

## Conclusions

For the first time, all of the RNA isolated from an infected tree have been used in parallel to sequence viroid species and small non coding RNA species. On the one hand, this work led to the determination of 18 novel PLMVd variants. On the other hand, the use of high throughput sequencing provided the largest library of siRNA reported yet for a viroid infection. It was shown that the entire PLMVd genome is retrieved in the siRNA population. Moreover, PLMVd strands of both polarities appeared susceptible for targeting, although specific regions for each one are over-represented in terms of siRNAs. Those regions, which are not the same for each polarity, do not necessarily correlate with double-stranded regions that could be substrates for Dicer-like enzymes. The nature of the first 5' nucleotide in the 21-22 nt population revealed a bias toward a C or a U in viroid-derived siRNAs. In fact this bias is also observed in the non-PLMVd-siRNAs. Unlike the infected library, the majority of the healthy sequences has A or G at their 5'. Importantly, the large libraries of siRNAs created in this study led to several observations concerning the silencing mechanism associated with PLMVd infection.

At the same time this manuscript was being submitted, another study describing deep sequencing of small RNAs associated to PLMVd infection was released [31]. The authors have showed that PLMVd of both polarities can generate predominantly small 21-22 nt long RNAs *in vivo*. These small RNAs map the PLMVd genome adopting a hotspot profile which is different from one polarity to the other (plus *vs *minus), although their libraries of PLMVd-associated siRNAs were smaller. However, the Di Serio et al. study has the advantage of reporting data isolated from two peach plants. The present study offers two important differences: Firstly, the fact that the sequencing of the small ncRNAs was also performed on a healthy plant ensures that there are no species showing homologies with PLMVd. Secondly, the PLMVd sequences used to identify viroid-associated siRNAs were obtained from the same sample as the small RNAs instead of using sequences reported several years ago [31]. It has been shown that PLMVd is a quasi-species and infection using a plasmid was sufficient for the observation of several sequences in a single plant [32]. There is another recent study that includes high throughput sequencing of small ncRNAs from grapevine infected by hop stunt viroid and grapevine yellow speckle viroid 1, two viroids from the other family (i.e. *Pospiviroidae *family) [33]. Clearly, high-throughput sequencing has become the method of choice for the identification of viroid-associated siRNAs.

## Methods

### RNA extraction and PLMVd analysis

Total RNA was extracted from the leaves of peach (Siberian C cultivar) and nectarine trees grown on the West Coast of Canada (Sidney, British Columbia) using Trizol (Invitrogen) according to the manufacturer's instructions. PLMVd can infect nectarine as well as peach trees [34]. The sample from the nectarine plant should be considered as a negative control in which there is no direct comparison of the ncRNA library with the one from the PLMVd-infected peach plant. These RNA samples were quantified by UV spectroscopy, and their qualities verified by 1% agarose gel electrophoresis.

For PLMVd cloning, 5 μg of total RNA were incubated in the presence of 3 U of RQ1 DNAse (Promega) at 37°C for 1 h in a total volume of 20 μL containing 40 mM Tris-HCl pH 8.0, 10 mM MgSO_4 _and 1 mM CaCl_2_. Following two phenol-chloroform extractions and an ethanol precipitation, the RNA was reverse transcribed for 1 h at 45°C in the presence of the reverse primer 5'-AGTGCTCCGAATAGGGCACCCCAAGGTGG-3' using SuperScript III (Invitrogen) in 20 μL reactions as recommended by the manufacturer. Upon completion of the reverse transcription reactions, 10 μg of RNase A were added and the mixtures incubated for 15 min at room temperature. Ten microliters of the reverse transcriptase reactions were then used in PCR amplification reactions performed using purified *Pfu *DNA polymerase. The reactions were carried out in a final volume of 100 μL containing 2 μM of each DNA primer, 2 mM MgSO_4_, 200 μM dNTP, 10 mM Tris-HCl (pH 8.85), 25 mM KCl and 5 mM (NH_4_)_2_SO_4_. The reactions were incubated at 94°C for 1 min prior to the addition of the enzyme, and were then subjected to 25 amplification cycles (30 sec at 94°C, 60 sec at 50°C and 30 sec at 72°C). The reverse primer was the same as that used previously in the reverse transcription reactions, while the forward primer was 5'-GCAGTTCCCGATAGAAAGGCTAAGCACC-3'. The amplifications were analyzed by loading 10 μL aliquots on a 1% agarose gel. The gel band corresponding to a fragment of 338 nt in length was cut out and the DNA extracted using Spin-X columns (Corning Science Products). The resulting DNA was then incubated in the presence of *Taq *DNA polymerase (New England Biolabs) for 20 min at 72°C and ligated into pGEM-T vector (Promega). After transformation into *E.coli*, 23 clones were sequenced at the Platefome de séquençage et de génotypage des genomes (CRCHUL) (Québec, Canada). All sequences were deposited to NCBI, accession numbers GQ499305 to GQ499322.

### Isolation of small RNAs and high-throughput sequencing

For each library (healthy and PLMVd-infected) 10 μg of total RNA was size fractionated on a 15% denaturing polyacrylamide gel and the 18-30 nt fraction excised from the gel. Following elution of the RNA, 5' and 3' adapters were added to the RNA species using the Digital Gene Expression for Small RNA Sample Prep Kit (Illumina). After reverse transcription, the samples were PCR amplified, purified on 10% polyacrylamide gels and then eluted according to the manufacturer's protocol. High-throughput Illumina Solexa sequencing was performed at The Center for Applied Genomics (Toronto, Canada).

Unfiltered sequence reads from both sets were trimmed of the 3' adapter. Reads that did not contain the 3' linker, and those shorter than 16 nt, were discarded. The resulting reads were aligned on the PLMVd sequences, as well as on other control RNA sequences, using the Exonerate software [19]. No mismatches were permitted in the pairwise sequence comparison. All small RNA reads are available at the GEO database with accession number GSE18764.

## Authors' contributions

J-PP conceived the study. PS-P isolated and cloned small RNAs that had been sent to Solexa sequencing. CH cloned the new PLMVd variants and contributed to bioinformatic analysis. FB conceived and performed the bioinformatic analysis. J-PP and FB wrote the manuscript. All authors read and approved the final manuscript.

## Supplementary Material

Additional file 1 New PLMVd sequences and their NCBI accession number. Click here for file
