# Supplementary material for: In-depth sequencing of the siRNAs associated with peach latent mosaic viroid infection
Source: BMC Mol Biol. 2010 Feb 16;11:16. doi: 10.1186/1471-2199-11-16 (PMC2830927; doi:10.1186/1471-2199-11-16)
Supplement: Additional file 1 — New PLMVd sequences and their NCBI accession number. [file 1471-2199-11-16-S1.PDF]

Additional file1
New PLMVd sequences and their NCBI accession number..

>GQ499305
ACUCAUAAGUUUCGUCACAUCUCAGUGACUCAUCAGUGGGCUUAGCCCAGACUUAUGAG
AGAAUAAGACCUCUCAGCCCCUCCACCUUGGGGUGCCCUAUUCGGAGCACUGCAGUUCC
CGAUAGAAAGGCUAAGCACGUCGCAUUGACGUAAGGUGGGACGUUUCCCUAGGGUUUGA
GCGGUCGAACCCAGGGGGAGUGUGACCCAGGUACCGCCGUAGAAACUGGGUUACGACGC
CUACCCGGGAUUCAAACCCGGUCCCCCUCAGAGGUGACUCUGAGUGAAAGGUCUGCGCU
AAGCGCACUGAUGAGUUCCUGAAAUGGAACGAAACCUUUUGAA

>GQ499306
AUCCAUAAGUUUCGUCGCAUCCCAGCGACUCGUCAGUGGGCUAAGCCCAGACUUAUGAG
AGAGUGAUUACCUCUCAGCCCCUCCACCUUGGGGUGCCCUAUUCGGAGCACUGCAGUUC
CCGAUAGAAAGGCUAAGCACGUCGCAAUGACGUAAGGUGGGACUUUUUCCCUAGGGUUC
GAGCGGUCGAACCCAGGGGGAGUGUGAUCCAGGUACCGCCGUAGAAACUGGAUUACGAC
GUCUACCCGGGAUUCAAACCCGGUCCCCCUCAGAGGUGACUCUGAGUGAAAGGUCUGUG
CUUAGCACACUGACGAGUUCCUGAAAUGGAACGAAACCUUUCUA

>GQ499307
ACCCAUAAGUUUCGUCGCAUCCCAGCGACUCGUCAGUGGGCUAAGCCCAGACUUAUGAG
AGAGUGGUUACCUCUCAGCCCCUCCACCUUGGGGUGCCCUAUUCGGAGCACUGCAGUUC
CCGAUAGAAAGGCUAAGAACGUCGCAAUGACGUAAGGUGGGACUUUUCCCUAGGGUUCG
AGCGGUCGAACCCAGGGGGAGUGUGAUCCAGGUACCGCCGUAGAAACUGGAUUACGACG
UCUACCCGGGAUUCAAACCCGGUCCCUCUCAGAGGUGACUCUGAGUGAAAGGUCUGUGC
UUAGCACACUGACGAGUUCCUGAAAUGGAACGAAACCUUUAUA

>GQ499308
ACCCAUAAGUUUCGUCGCAUCCCAGCGACUCGUCAGUGGGCUAAGCCCAGACUUAUGAG
AGAGUGGUUACCUCUCAGCCCCUCCAUCUUGGGGUGCCCUAUUCGGAGCACUGCAGUUC
CCGAUAGAAAGGCUAAGAACGUCGCAAUGACGUAAGGUGGGACUUUUCCCUAGGGUUUG
AGCGGUCGAACCCAGGGGGAGUGUGAUCCAGGUACCGCCGUAGAAACUGGAUUACGACG
UCUACCCGGGAUUCAAACCCGGUCCCUCUCAGAGGUGACUCUGAGUGAAAGGUCUGUGC
UUAGCACACUGACGAGUUCCUGAAAUGGAACGAAACCUUUGUA

>GQ499309
AUCCAUAAGUUUCGUCGCAUCCCAGCGACUCGUCAGUGGGCUAAGCCCAGACUUAUGAG
AGAGUGGUUACCUCUCAGCCCCUCCACCUUGGGGUGCCCUAUUCGGAGCACUGCAGUUC
CCGAUAGAAAGGCUAAGCACGUCGCAAUGACGUAAGGUGGGAUUUUUCCCUAGGGUUUG
AGCGGUCGAACCCAGGGGGAGUGUGAUCCAGGUACCGCCGUAGAAACUGGAUUACGACG
UCUACCCGGGAUUCAAACCCGGUCCCCCUCAGAGGUGACUCUGAGUGAAAGGUCUGUGC
UUAGCACACUGACGAGUUCCUGAAAUGGAACGAAACCUUUCUA

>GQ499310
ACCCAUAAGUUUCGUCGCAUCCCAGCGACUCGUCAGUGGGCUAAGCCCAGACUUAUGAG
AGAGUGGUUACCUCUCAGCCCCUCCACCUUGGGGUGCCCUAUUCGGAGCACUGCAGUUC
CCGAUAGAAAGGCUAAGAACGUCGCAAUGACGUAAGGUGGGACUUUUCCCUAGGGUUUG

AGCGGUCGAACCCAGGGGGAGUGUGAUCCAGGUACCGCCGUAGAAACUGGAUUACGACG
UCUACCCGGGAUUCAAACCCGGUCCCUCUCAGAGGUGACUCUGAGUGAAAGGUCUGUGC
UUAGCACACUGACGAGUUCCUGAAAUGGAACGAAACCUUUGUA

>GQ499311
ACCCAUAAGUUUCGUCGCAUCCCAGCGACUCGUCAGUGGGCUAAGCCCAGACUUAUGAG
AGAGUGGUUACCUCUCAGCCCCUCCACCUUGGGGUGCCCUAUUCGGAGCACUGCAGUUC
CCGAUAGAAAGGCUAAGAACGUCGCAAUGACGUAAGGUGGGAUUUUUCCCUAGGGUUUG
AGCGGUCGAACCCAGGGGGAGUGUGAUCCAGGUACCGCCGUAGAAACUGGAUUACGACG
UCUACCCGGGAUUCAAACCCGGUCCCCCUCAGAGGUGACUCUGAGUGAAAGGUCUGUGC
UUAGCACACUGACGAGUUCCUGAAAUGGAACGAAACCUUUAUA

>GQ499312
AUCAAAAAGUUUCGUCGCAUCUCAGCGACUCAUCAGUGGGCUAAGCCCAGACUUAUGAG
AGAAGUGAUGACCUCUCAGCCCCUCCACCUUGGGGUGCCCUAUUCGGAGCACUGCAGUU
CCCGAUAGAAAGGCUAAGCACGUCGCAAUGACGUAAGGUGGGAUUUUUCCCUAGGGUUU
GAGCGGUCGAACCCAGGGGGAGUGUGAUCCAGGUACCGCCGUAGAAACUGGAUUACGAC
GCCUACCCGGGAUUCAAACCCGGUCCCCCUCAGAGGUGACUCUGAGUGAAAGGUCUGUG
CUUAGCACACUGAUGAGUUCCUGCAAUGGAACGAAACCUUUCUC

>GQ499313
ACCCAUAAGUUUCGUCGCAUCCCAGCGACUCGUCAGUGGGCUAAGCCCAGACUUAUGAG
AGAGUGGUUACCUCUCAGCCCCUCCACCUUGGGGUGCCCUAUUCGGAGCACUGCAGUUC
CCGAUAGAAAGGCUAAGAACGUCGCAAUGACGUAAGGUGGGAUUUUUCCCUAGGGUUUG
AGCGGUCGAACCCAGGGGGAGUGUGAUCCAGGUACCGCCGUAGAAACUGGAUUACGACG
UCUACCCGGGAUUCAAACCCGGUCCCCCUCAGAGGUGACUCUGAGUGAAAGGUCUGUGC
UUAGCACACUGACGAGUUCCUGAAAUGGAACGAAACCUUUGUA

>GQ499314
AUCCAUAAGUUUCGUCGCAUCCCAGCGACUCGUCAGUGGGCUAAGCCCAGACUUAUGAG
AGAGUGAUUACCUCUCAGCCCCUCUACCUUGGGGUGCCCUAUUCGGAGCACUGCAGUUC
CCGAUAGAAAGGCUAAGAACGUCGCAAUGACGUAAGGUGGGAUUUUUCCCUAGGGUUUG
AGCGGUCGAACCCAGGGGGAGUGUGAUCCAGGUACCGCCGUAGAAACUGGAUUACGACG
UCUACCCGGGAUUCAAACCCGGUCCCCCUCAGAGGUGACUCUGAGUGAAAGGUCUGUGC
UUAGCACACUGACGAGUUCCUGAAAUGGAACGAAACCUUUCUA

>GQ499315
ACUCAUAAGUUUCGUCGCAUCCCAGCGACUCAUCAGUGGGCUUAGCCCAGACUUAUGAG
AGAAUUAGUCACCUCUCAGCCCCUCCACCUUGGGGUGCCCUAUUCGGAGCACUGCAGUU
CCCGAUAGAAAGGCUAAGAACGUCGCAAUGACGUAAGGUGGGACUUUUCCCUAGGGUUU
GAGCGGUCGAACCCAGGGGGAGUGUGAUCCAGGUACCGCCGUAGAAACUGGAUUACGAC
GUCUACCCGGGAUUCAAACCCGGUCCCUCUCAGAGGUGACUCUGAGUGAAAGGUCGUGC
UUAGCACACUGACGAGUUCCUGAAAUGGAACGAAACCUUUGUG

>GQ499316
ACCCAUAAGUUUCGUCGCAUCCCAGCGACUCGUCAGUGGGCUAAGCCCAGACUUAUGAG
AGAGUGGUUACCUCUCAGCCCCUCCACCUUGGGGUGCCCUAUUCGGAGCACUGCAGUUC

CCGAUAGAAAGGCUAAGAACGUCGCAAUGACGUAAGGUGGGACUUUUCCCUAGGGUUUG
AGCGGUCGAACCCAGGGGGAGUGUGAUCCAGGUACCGCCGUAGAAACUGGAUUACGACG
UCUACCCGGGAUUCAAACCCGGUCCCCCUCAGAGGUGACUCUGAGUGAAAGGUCUGUGC
UUAGCACACUGACGAGUUCCUGAAAUGGAACGAAACCUUUCUG

>GQ499317
ACCCAUAAGUUUCGUCGCAUCCCAGCGACUCGUCAGUGGGCUAAGCCCAGACUUAUGAG
AGAGUGGUUACCUCUCAGCCCCUCCACCUUGGGGUGCCCUAUUCGGAGCACUGCAGUUC
CCGAUAGAAAGGCUAAGCACCUCGCAAUGAGGUAAGGUGGGACUUUUCCUUCGGGAACC
AAGCGGUUGGUUCCGAGGGGGGUGUGAUCCAGGUACCGCCGUAGAAACUGGAUUACGAC
GUCUACCCGGGAUUCAAACCCGGUCCCCCUCAGAGGUGACUCUGAGUGAAAGGUCGUGC
UUAGCACACUGACGAGUUCCUGAAAUGGAACGAAACCUUUAUG

>GQ499318
AUCCAUAAGUUUCGUCGCAUCCCAGCGACUCGUCAGUGGGCUAAGCCCAGACUUAUGAG
AGAGUGAUUACCUCUCAGCCCCUCCACCUUGGGGUGCCCUAUUCGGAGCACUGCAGUUC
CCGAUAGAAAGGCUAAGAACGUCGCAAUGACGUAAGGUGGGAUUUUUCCCUAGGGUUUG
AGCGGUCGAACCCAGGGGGAGUGUGAUCCAGGUACCGCCGUAGAAACUGGAUUACGACG
UCUACCCGGGAUUCAAACCCGGUCCCCCUCAGAGGUGACUCUGAGUGAAAGGUCGUGCU
UAGCACACUGACGAGUUCCUGAAAUGGAACGAAACCUUUCUA

>GQ499319
ACCCAUAAGUUUCGUCGCAUCCCAGCGACUCGUCAGUGGGCUAAGCCCAGACUUAUGAG
AGAGUGGUUACCUCUCAGCCCCUCCACCUUGGGGUGCCCUAUUCGGAGCACUGCAGUUC
CCGAUAGAAAGGCUAAGAACGUCGCAAUGACGUAAGGUGGGAUUUUUCCCUAGGGUUUG
AGCGGUCGAACCCAGGGGGAGUGUGAUCCAGGUACCGCCGUAGAAACUGGAUUACGACG
UCUACCCGGGAUUCAAACCCGGUCCCCCUCAGAGGUGACUCUGAGUGAAAGGUCUGUGC
UUAGCACACUGACGAGUUCCUGAAAUGGAACGAAACCUUUUUG

>GQ499320
ACUCAUAAGUUUCGUCGCAUCCCAGCGACUCGUCAGUGGGCUAAGCCCAGACUUAUGAG
AGAGUGGUUACCUCUCAGCCCCUCCACCUUGGGGUGCCCUAUUCGGAGCACUGCAGUUC
CCGAUAGAAAGGCUAAGCACGUCGCUUUGACGUAAGGUGGGACUUUUCCCUAGGGUUUG
AGCGGUCGAACCCAGGGGGAGUGUGACCCAGGUACCGCCGUAGAAACUGGGUUACGACG
CCUACCCGGGAUUCAAACCCGGUCCCCCUCAGAGGUGACUCUGAGUGAAAGGUCUGCGC
UAAGCGCACUGAUGAGUUCCUGAAAUGGAACGAAACCUUUUUA

>GQ499321
ACUCAUAAGUUUCGUCGCAUCCCAGCGACUCAUCAGUGGGCUUAGCCCAGACUUAUGGG
AGAUUAGUUACCUCUCAGCCCCUCCACCUUGGGGUGCCCUAUUCGGAGCACUGCAGUUC
CCGAUAGAAAGGCUAAGCACGUCGCAUUGACGUAAGGUGGGACGUUUCCCUAGGGUUUG
AGCGGUCGAACCCAGGGGGAGUGUGACCCAGGUACCGCCGUAGAAACUGGGUUACGACG
CCUACCCGGGAUUCAAACCCGGUCCCCCUCAGAGGUGACUCUGAGUGAAAGGUCGCGCU
AAGCGCACUGAUGAGUUCCUGAAAUGGAACGAAACCUUUGAUA

>GQ499322

AUCCAUAAGUUUCGUCGCAUCCCAGCGACUCGUCAGUGGGCUAAGCCCAGACUUAUGAG
AGAGUGGUUACCUCUCAGCCCCUCCACCUUGGGGUGCCCUAUUCGGAGCACUGCAGUUC
CCGAUAGAAAGGCUAAGCACGUCGCAAUGACGUAAGGUGGGACUUUUCCCUAGGGUUUG
AGCGGUCGAACCCAGGGGGAGUGUGAUCCAGGUACCGCCGUAGAAACUGGAUUACGACG
UCUACCCGGGAUUCAAACCCGGUCCCCCUCAGAGGUGACUCUGAGUUAAAGGUCUGUGC
UUAGCACACUGACGAGUUCCUGAAAUGGAACGAAACCUUUCUA
